# ExoFiT trial at the Atacama Desert (Chile): Raman detection of biomarkers by representative prototypes of the ExoMars/Raman Laser Spectrometer

**DOI:** 10.1038/s41598-021-81014-z

**Published:** 2021-01-14

**Authors:** Marco Veneranda, Guillermo Lopez-Reyes, Jesus Saiz, Jose Antonio Manrique-Martinez, Aurelio Sanz-Arranz, Jesús Medina, Andoni Moral, Laura Seoane, Sergio Ibarmia, Fernando Rull

**Affiliations:** 1 Department of Condensed Matter Physics, Crystallography and Mineralogy, Univ. of Valladolid, Spain, Ave. Francisco Vallés, 8, 47151 Boecillo, Spain; 2 National Institute for Aerospace Technology (INTA), Torrejón de Ardoz, Spain

**Keywords:** Planetary science, Astrobiology, Mineralogy, Analytical chemistry

## Abstract

In this work, the analytical research performed by the Raman Laser Spectrometer (RLS) team during the ExoFiT trial is presented. During this test, an emulator of the *Rosalind Franklin* rover was remotely operated at the Atacama Desert in a Mars-like sequence of scientific operations that ended with the collection and the analysis of two drilled cores. The in-situ Raman characterization of the samples was performed through a portable technology demonstrator of RLS (RAD1 system). The results were later complemented in the laboratory using a bench top RLS operation simulator and a X-Ray diffractometer (XRD). By simulating the operational and analytical constraints of the ExoMars mission, the two RLS representative instruments effectively disclosed the mineralogical composition of the drilled cores (k-feldspar, plagioclase, quartz, muscovite and rutile as main components), reaching the detection of minor phases (e.g., additional phyllosilicate and calcite) whose concentration was below the detection limit of XRD. Furthermore, Raman systems detected many organic functional groups (–C≡N, –NH_2_ and C–(NO_2_)), suggesting the presence of nitrogen-fixing microorganisms in the samples. The Raman detection of organic material in the subsurface of a Martian analogue site presenting representative environmental conditions (high UV radiation, extreme aridity), supports the idea that the RLS could play a key role in the fulfilment of the ExoMars main mission objective: to search for signs of life on Mars.

## Introduction

Led by ESA with the collaboration of Roscosmos, the ExoMars 2022 rover mission will pursue the detection of signs of present or past life on Mars^1,2^. To achieve this goal, the designed payload of the *Rosalind Franklin* rover will employ a set of panoramic instruments (PANCAM^3^ and ISEM^4^) to explore the surrounding environment, thus providing crucial data to be used in the navigation of the rover and in the identification of areas of high scientific interest. A ground-penetrating RADAR (WISDOM^5^) and a passive neutron spectrometer (ADRON-RM^6^) will investigate the subsurface, helping in the selection of potential drilling places. The ExoMars Drill Unit^7^ (hosting the MA_MISS visible and near infrared spectrometer^8^) will collect geologic samples down to a depth of 2 m, thus accessing material that have been sheltered from UV Radiation and further alteration processes. CLUPI^9^ will provide textural information from the sampled materials through the collection of high-resolution images, while the sample preparation and distribution system (SPDS) will crush the materials and deliver the powders to the analytical laboratory of the rover^10^. Here, the visible/near-infrared spectrometer (MicrOmega^11^) and the Raman Laser Spectrometer (RLS^12^) will perform coordinated analyses^13^ to identify the mineralogical composition of the samples and to reveal the potential presence of biomarkers. Spectroscopic results will be used to select the optimal scientific targets to be delivered to MOMA (Mars Organic Molecule Analyzer system), that will extract and analyse the organic molecules potentially preserved within the mineralogical matrix^14^.

Apart from the technical and engineering challenges that meant the development of the mentioned instruments, the success of the mission also relies on the complex coordination work required for their remote control and synergic management Recognizing the need for training the ExoMars teams and enhancing collaboration practices between instrument working groups, ESA organized the ExoMars-like Field Testing (ExoFiT) trials^15^, the second of which was carried out at the Atacama Desert (Chile) in February 2019. In addition to presenting a Martian-like desertic landscape, the presence of extremophile microorganisms populating Atacama’s subsurface made this the ideal location to test the ability of the rover’s payload to detect biomarkers in this kind of environments^16^.

During the trial, an emulator of the *Rosalind Franklin* rover (Charlie) was used to perform a complex sequence of scientific and engineering operations (from descending the landing platform to collecting drill cores) following the ExoMars Reference Surface Mission (RSM)^17^. During the mission simulation, the LCC team (Local Control Centre, located at the Atacama Desert, near the ESA Paranal Observatory) manoeuvred the rover and managed the acquisition and upload of the collected data. From 11,000 km of distance, the RCC team (Remote Control Centre, located at the European Centre for Space Applications and Telecommunications, UK) simulated the operations on Mars, planning the different activities for the next sol by only relying on the data returned by the rover^18^.

As part of the LCC team, science and engineering roles were covered by personnel from the University of Valladolid (UVa) and the National Institute for Aerospace Technology (INTA), who carried out the Raman characterization of the subsoil cores drilled by the rover. The Raman characterization was done using two spectrometers. A first mineralogical evaluation of the samples was performed using the RAD1 system (RAman Demonstrator 1), which is a portable RLS technology demonstrator assembled by the RLS team to carry out in-situ analyses in terrestrial analogue sites^19^. The RAD1 spectrometer has similar range of analysis (70–4200 cm^−1^), laser wavelength (532 nm) and power output (7 mW on the sample), spot of analysis (≈ 50 µm) and spectral resolution (6–10 cm^−1^) to the RLS, providing spectra qualitatively comparable to those soon gathered on Mars. Afterwards, more detailed spectroscopic analyses were carried out in the laboratory by means of the RLS ExoMars Simulator, which characteristics have been described elsewhere^20^. As detailed in previous works, this is the optimal instrument to predict the potential scientific outcome of the RLS flying model^21,22^. Indeed, in addition to the RLS-like optical spectral characteristics (as the RAD1), the spectrometer is coupled to a replicate of the ExoMars/SPDS, and integrates the same algorithms developed for the RLS to perform the automatic multi-point analysis of Martian samples (e.g. Signal to Noise Ratio optimization, florescence quenching and acquisition parameters selection^23^). Raman spectra from the ExoFiT exercise were obtained under the same operational constraints of the rover, and were finally compared to XRD data, being this the reference instrument for the mineralogical study of geological samples.

Recognizing the scientific and logistic value of this mission simulation, the present work aims to (1) summarize the preliminary analytical results obtained by the RLS team from the study of Atacama Desert samples, (2) evaluate advantages and disadvantages provided by the use of the RLS representative prototypes in ExoMars-related studies, and (3) extrapolate valuable information about the potential role the RLS could play in the fulfilment of the ExoMars mission objectives.

## Materials and methods

### Atacama desert (Chile)

The Atacama Desert is a high plain covering an area of more than 100,000 km^2^ between northern Chile and southern Peru. The hyper arid climate of this region, persisting unchanged for the last 10 million years, is due to the concurrence of the foehn effect (triggered by the Andean Mountains), the Humboldt current and high-pressure atmospheric conditions (caused by Pacific anticyclones)^24^. The ExoFiT trial was carried out in the region of Antofagasta, about 11 km west of the ESO Paranal observatory (altitude of 2200 m). According to previous studies, three kinds of rocks dominate the mineralogy of this area: granodiorites (white–pink colour) and andesite (dark green) are composed of plagioclase and quartz in different concentration ratio, while gabbros (dark-gray color) contain feldspar and amphibole/pyroxene minerals^25^. In addition to these primary minerals, alteration products such as phyllosilicates and oxides (e.g. hematite) can be found in the area together with evaporites (nitrates, sulphates and chlorides).

Based on the data collected at the ESO Paranal observatory, this area presents high temperature oscillations (from − 8 to + 25 ºC), extremely low humidity values (5–20%) and an average annual rainfall below 10 mm^26^. In addition to the mentioned parameters, the extremely high levels of surface ultraviolet (UV) irradiance (> 1100 W/m^2^)^27^, make this area the perfect terrestrial analogue site to investigate the suitability of microbial life in extreme environments, similar to those that can be found on Mars and other planets^16^. Despite the harsh environmental conditions, extremophile microorganisms populate the subsurface of Atacama by relying on metabolic mechanisms that may have analogies with those that could be adopted in the shallow subsurface of Mars^28–30^. In light of the forthcoming deployment of Raman spectrometers on Mars (beside the RLS, Sherloc^31^ and SuperCam^32,33^ instruments onboard the NASA/Mars 2020 rover also need to be mentioned), Vitek et al. published several works using Atacama rocks and soil samples to assess the capability of this technique to detect biomarkers, gathering encouraging results^34–36^.

### Rover activity and samples collection

**Figure 1:**
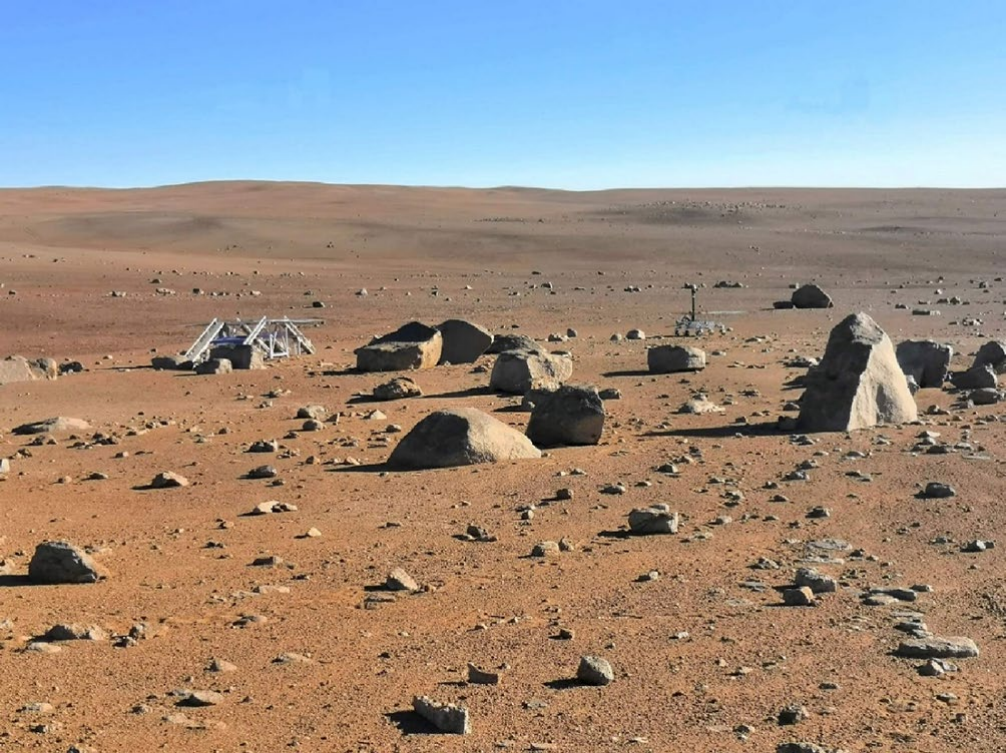
Image of the desertic area selected for the ExoFiT trial (Atacama Desert). The ExoMars rover and landing platform emulators can be observed.

As can be seen in Fig. 1, the Martian-like landscape of the area selected by the LCC team presents a reddish desertic pavement made of gravel, boulders and interspersed sand patches. The area also features small clay deposits and salt crusts, being these units of great astrobiological interest. Besides site selection, the LCC team took care of manoeuvring the rover and operating the whole set of ExoMars instruments accordingly to the commands received from mission control. As mission coordinator, the RCC made an assessment of the landing site, planning the descent from the landing platform and the driving through a safe route to reach an area of scientific interest. The subsurface stratigraphy of the selected site was then analysed by WISDOM (scan grid of approximately 5 * 5 m). Based on subsurface radar results, RCC selected the optimal drilling site. After drilling, the extracted soil core was imaged by CLUPI, separated in two samples (upper part UP and lower part LP) and sent for Raman analysis. During the ExoFiT trial, two experiment cycles were conducted, giving a total of two cores and four subsamples in total. Since the granulometry of the sample affects the quality of Raman results^37^ core samples were crushed and sieved to obtain a grain size distribution resembling the one prepared by the ExoMars/SPDS. The resulting samples were then placed into a replicate of the ExoMars sample holder and analysed by Raman.

### Instruments

For the in-situ characterization of drill cores, Raman analyses were performed directly at the analogue site and by following the time constraint imposed by the mission simulation. To do so, the RAD1 spectrometer was used. Assembled by the ERICA group, this portable instrument is composed of a commercial excitation laser source of 532 nm, a high resolution Thermo Electrically (TE) Cooled CCD Array spectrometer (2168 × 512 pixels) and a high line density diffraction grating (1800 lines per mm). The instrument was optically harnessed by optical fibers to a microscope with a 50 × objective, reproducing the analytical footprint of RLS (50 µm). The time constrains applicable to these analyses during ExoFiT test limited the time per sample to 1–1.5 h, a timeframe shorter than the nominal ExoMars rover operations.

Complementary analyses were done at the laboratory using the RLS ExoMars Simulator, a system with similar spectroscopic performance to RAD1, but incorporating the automatic operation capabilities of RLS. The instrument includes a continuous green excitation laser (532 nm), a high resolution TE Cooled CCD Array spectrometer and an optical head with a long working distance objective of 50x. The instrument is coupled to three axis micrometric positioning system with a refillable container (emulating the ExoMars sample holder) that allows the definition of analysis rasters on the sample. Software-wise, the RLS ExoMars Simulator implements the same algorithms developed for the RLS^23^, allowing the automatic analysis of the samples auto adjusting the acquisition parameters. For both spectrometers, spectra acquisition was performed through a custom developed software based on LabVIEW 2013 (National Instruments), while the IDAT/SpectPro software was used for data processing and interpretation^38^. Knowing that the quantum efficiency of CCD detectors varies with the wavelength, the intensity of all spectra was corrected by following the method presented by Sanz Arranz et al.^39^ Besides Raman analyses, the mineralogical characterization of powdered materials was complemented by XRD data. For this purpose, a laboratory Discover D8 XRD (Bruker) was used. The diffractometer is composed of a Cu X-ray excitation source (wavelength 1.54 Å) and a LynxEye detector. Fine-powdered rocks (granulometry ≤ 150 µm) were analysed by setting a scan range between 5 and 70° 2θ, a step increment in 2θ of 0.01 and a count time of 0.5 s per step. The collected diffractograms were interpreted using the BRUKER DIFFRAC.EVA software.

## Results

### RAD 1

Drilled cores were analysed in-situ by using the portable RAD1 spectrometer. For this purpose, powdered samples were placed in a replicate of the ExoMars sample holder and, after flattening, a raster of measurements was performed by moving the X positioner at regular intervals of ≈ 300 µm. For each spot of analysis, the acquisition parameters were optimized manually. It must be noted that in-situ analyses were hampered by meteorological conditions, since the strong wind blowing during the trial produced vibrations to the spectrometer, compromising the acquisition of many Raman spectra. For this reason, a very limited number of spectra per sample (between 4 and 6) could be collected within the time constraints imposed by the mission simulation.

**Figure 2:**
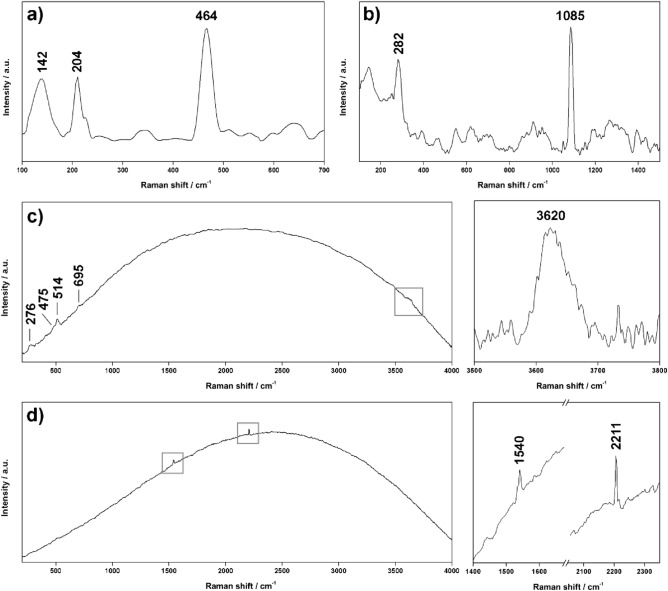
Characteristic Raman spectra of (**a**) quartz, (**b**) calcite, (**c**) mixed feldspar-mica, and (**d**) organics, collected in-situ by the RAD1 system (532 nm). The baseline of spectra a and b has been corrected using dedicated IDAT/SpectPro tools.

Despite the limited amount of Raman data, different mineral phases were successfully detected. Starting from ADC2 drill core, quartz (SiO_2_, 142, 204 and 464 cm^−1^, Fig. 2a) was detected in both UP (upper part) and LP (lower part) subsamples. Calcite (CaCO_3_) was also detected in the upper part of the core (Fig. 2b), while the weak band found at 660 cm^−1^ could be assigned to amphibole minerals. As displayed in Fig. 2d, one of the analysed spots of sample ADC2-LP returned peaks at 1540 and 2211 cm^−1^. Assuming that those signals are emitted by organics, the peak at 1540 cm^−1^ can be assigned to the absorption of the asymmetric stretching of C–(NO2) from aromatic nitro compounds^40^, while signals between 2210 and 2225 cm^−1^ can be associated with the stretching of nitrile compounds (C≡N, probably conjugated with C=O groups^41^).

On the other hand, Raman analysis of sample ACD2-LP displayed peaks at 276, 475 and 514 cm^−1^, revealing the presence of feldspar (Fig. 2c). However, the mineral phase within this group was not clearly identified due to the low Signal to Noise Ratio (SNR) of the obtained spectra. Besides feldspar, the same spectra displayed a broad band at 3600 cm^−1^ (vibration of –OH group), together with an additional minor signal at 695 cm^−1^. According to the work published by Wang et al., 2015^42^, these signals are characteristic of phyllosilicates within the mica subgroup (probably muscovite).

In-situ Raman analysis of ADC1 core were quite inconsistent since the two subsamples were highly fluorescent, which is a side-effect from electronic excitation that increases the background signal in the spectra, masking mineral Raman bands. Although the long exposure of the spot to the excitation laser helps quenching the florescence, this operation could not be performed due to the abovementioned vibrations induced by the wind. Despite this limitation, feldspar minerals were effectively detected in both LP and UP subsamples.

### RLS ExoMars simulator

After the automatic adjustment of the spectra acquisition parameter to the constraints established for the ExoFiT trial, the number of Raman spectra automatically collected from each sample with this instrument varied between 9 and 12, which is below the minimum number of analysis per core that are expected to be carried out on Mars (20). This can be explained by the fact that the time dedicated to the in-situ study of drilled cores was narrowed due to logistic reasons (two RSM measurement cycles needed to be compressed within a time frame of 10 sols). However, additional spots were analysed to reach a total of 39 spectra per sample, being this the maximum number of analysis to be nominally performed on regular operations on Mars. The results described below are based on the complete set of Raman data gathered from each sample, although the summary provided in Table 1 allows one to distinguish the minerals detected within the ExoFiT-constrained time frame (black cross) from those additionally detected using nominal ExoMars mission parameters (red cross).

**Table 1:**
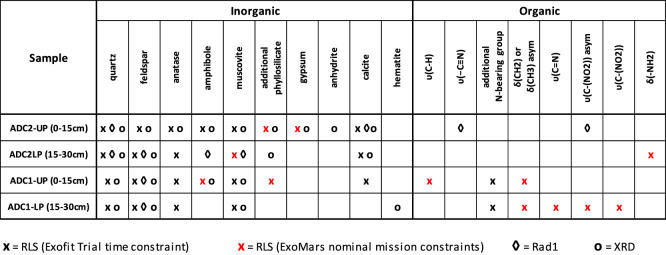
Summary of organic and inorganic phases detected through the analytical study of ADC1 and ADC2 drilled cores.

**Figure 3:**
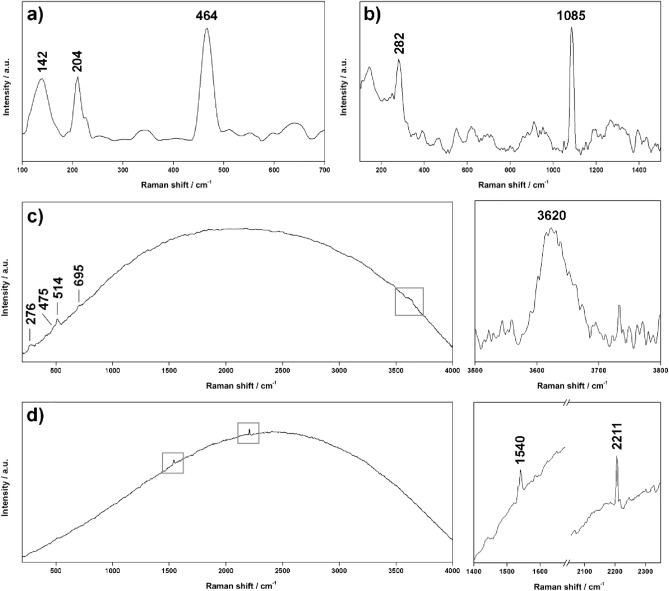
Characteristic Raman spectra of (**a**) quartz, (**b**) anatase, (**c**) plagioclase, (**d**) k-feldspar, (**e**) mica, (**f**) calcite, (**g**) hornblende and (**h**) gypsum, collected in the laboratory by the RLS ExoMars Simulator (532 nm). The baseline of spectra e, g and h has been corrected using dedicated IDAT/SpectPro tools. Raman signals proceeding from additional compounds are labelled with an asterisk.

Starting from the ADC2 core, both UP and LP subsamples showed Raman features from quartz (main peak at 464 cm^−1^ and secondary signals at 124, 202, 263, 354, 805 and 1159 cm^−1^, Fig. 3a), anatase (TiO_2_, main peaks at 142, 394, 510 and 634 cm^−1^, Fig. 3b) and feldspar. By comparing the vibrational profile of feldspar spectra, different mineral phases were identified. For example, the positions of the peaks detected in the spectrum shown in Fig. 3c (main signals at 478 and 508, together with minor peaks at 167, 286, 409, 566, 763, 809 and 1100 cm^−1^) matched perfectly with the Raman features from albite^43^, being this mineral the Na-rich end member of the plagioclase subgroup (NaAlSi_3_O_8_). As displayed in Fig. 3d, further spectra matched with anorthite reference spectrum (peaks at 150, 276, 401, 473, 515, 756, 799 and 1120 cm^−1^, confirming the additional presence of K-feldspars in both subsamples. Albite and anorthite spectra were found to be often associated with additional peaks at 264, 407, 702 and 3628 cm^−1^, which are consistent with the muscovite reference spectrum (KAl_2_(Si_3_Al)O_10_(OH)_2_, Fig. 3e). In addition to the mentioned mineral phases, calcium carbonate was additionally detected (main peaks at 149, 275, 709 and 1085 cm^−1^, Fig. 3f) in both subsamples.

**Figure 4:**
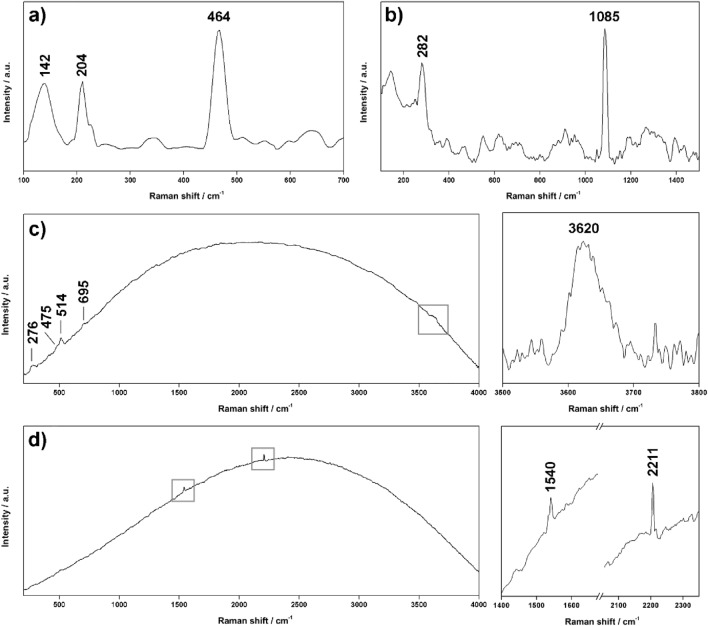
Characteristic Raman spectra of organic compounds collected in the laboratory by the RLS ExoMars Simulator (532 nm). All spectra have been smoothed using dedicated IDAT/SpectPro tools.

Raman results from ADC2-UP showed a higher and more complex mineralogical heterogeneity of this subsample when compared to AC2-LP. As shown in Fig. 3g, additional Raman peaks were found at 220 and 670 cm^−1^, matching the characteristic signals of amphibole minerals (probably actinolite, Ca_2_(Mg,Fe)_5_Si_8_O_22_ (OH)_2_). As can be seen in Fig. 3h, the detection of clear peaks at 411, 490, 620, 1008 and 1135 cm^−1^ revealed the presence of gypsum (Ca_2_SO_4_) as additional evaporitic mineral. One of the spectra gathered from the upper part of the core displayed two weak bands in the spectral range between 3600 and 3700 cm^−1^. In detail, the peak at 3630 cm^−1^ matches the mentioned –OH vibration from muscovite, while the signal at 3695 cm^−1^ could be associated with additional clay minerals such as kaolinite, serpentine or chlorite^42^. In addition to those, a broad band at 3425 cm^−1^ was found associated with two phyllosilicate spectra. When compared to the Raman emission of organic functional groups described elsewhere^40^, the detected signal matches the characteristic position of the in-phase bending mode of aromatic amines (–NH2, Fig. 4c).

Raman spectra from the laboratory analyses of both drill cores, ADC1 and ADC2, showed a similar mineral composition. Indeed, quartz, anatase, plagioclase and K-feldspar were found to be the main mineral components of both UP and LP subsamples, while calcite and actinolite were exclusively detected in the upper part of the core. Besides the detection of mineral phases, the RLS ExoMars Simulator could detect Raman features from potential biomarkers, as displayed in Fig. 4a. A doublet at 2190 and 2250 cm^−1^ was clearly identified in both UP and LP samples that, according to the results presented in previous works, could correspond to the vibration mode of different functional groups containing nitrogen^40^. Similarly, the peak observed around 1445 cm^−1^ can be either attributed to the symmetric bending of –CH2 or the asymmetric bending of –CH3. Further potential organic peaks were detected on sample UP, as shown in Fig. 4a, with features appearing at 2800 and 2850 cm^−1^ that fall within the C–H stretching region (2800–3100 cm^−1^)^44^. Concerning sample LP, additional vibrational features from organic functional groups are shown in Fig. 4b,d, with peaks detected in the range between 1300 and 1700 cm^−1^. More specifically, the three signals at 1340, 1380 and 1530 cm^−1^ can be assigned to the C–(NO2) functional group (both symmetric and asymmetric stretching), while the peak at 1644 cm^−1^ can be related to the stretching mode of C=N.

### X-Ray Diffraction

**Figure 5:**
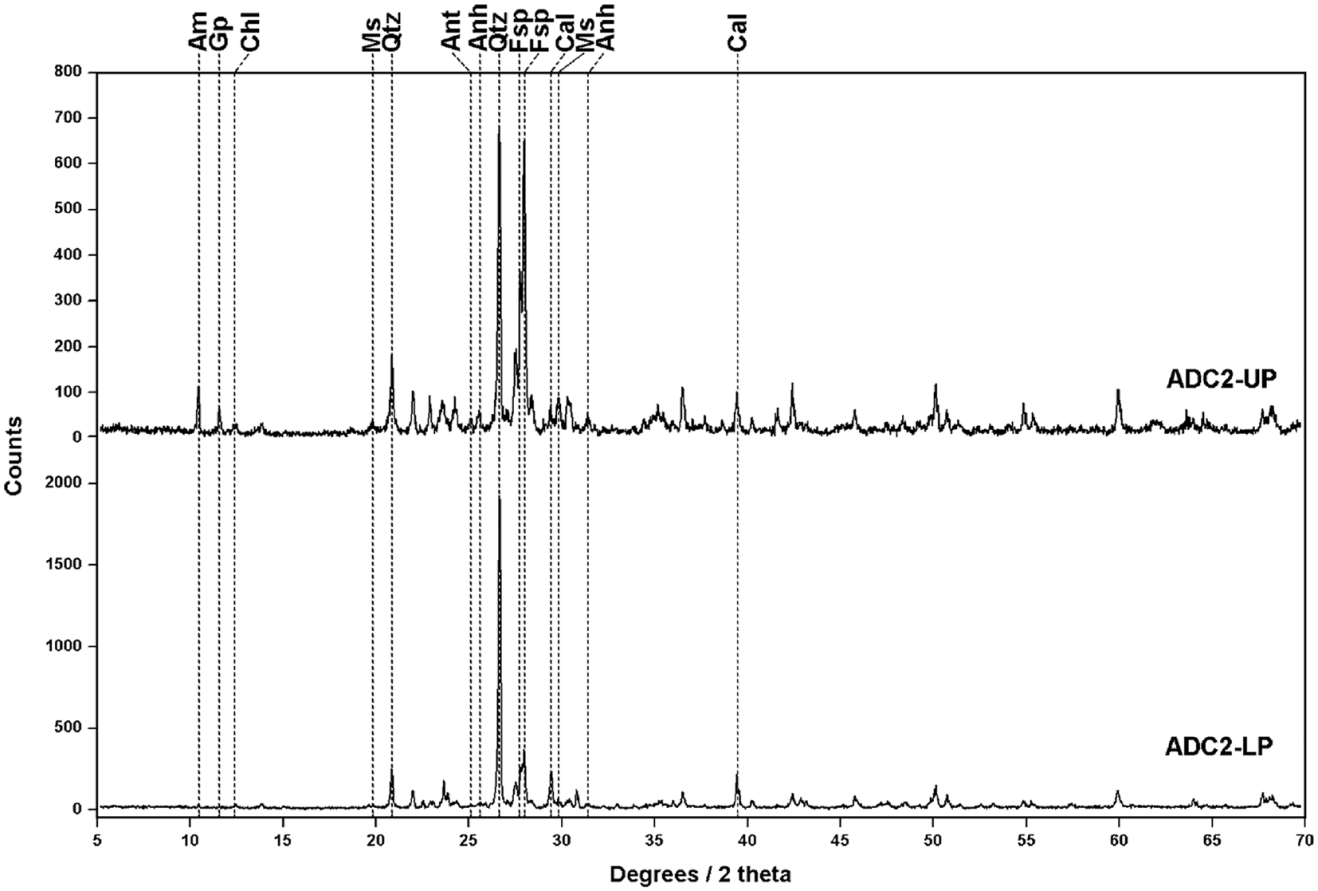
Diffractograms obtained from the analysis of samples ADC2-UP and -LP, revealing the presence of amphibole (Am, RUFF proxy ID: R110203), gypsum (Gy, R040029), chlorite (Chl, R060725), muscovite (Ms, R040104), quartz (Qtz, R040031), anatase (Ant, R060277), anhydrite (Anh, R040012), feldspar (Fsp, R040068) and calcite (Cal, R040070).

XRD is widely considered as the optimal technique to identify the crystalline phases of geological samples. As such, XRD analysis were carried out to have an objective confirmation of the results obtained by Raman spectroscopy. Starting from the ADC2 core, UP and LP subsamples (Fig. 5) returned very similar diffractograms, where quartz (based on 26.63 and 20.87 2θ values), plagioclase (27.74 and 27.93 2θ, matching albite patterns) and calcite (29.43 and 39.45 2θ) were found to be the main mineral phases in the powdered material. Additionally, the two diffractograms revealed additional peaks suggesting the presence of phyllosilicates. More specifically, both, muscovite (19.82 and 29.89 2θ) and chlorite (12.46 and 18.73 2θ, matching clinochlore pattern) were detected in the upper part of the core, while only chlorite was found in the lower part. UP sample also stood out for the detection of minor amounts of amphiboles (10.51 and 30.39 2θ, matching actinolite pattern), anhydrite (25.47 and 31.39 2θ), gypsum (11.58 2θ) and anatase (25.20 and 37.72 2θ).

**Figure 6:**
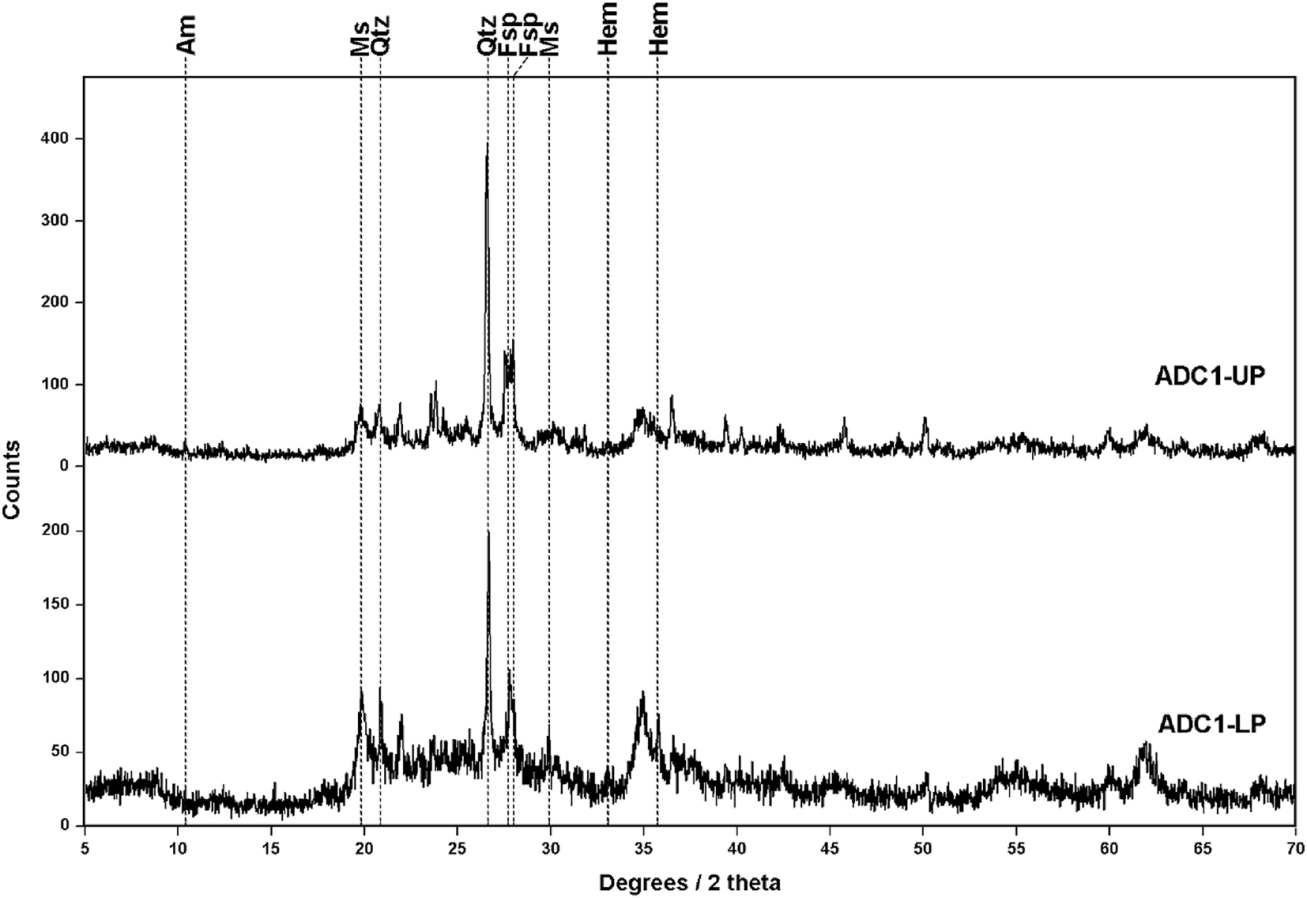
Diffractograms obtained from the analysis of samples ADC1-UP and -LP, revealing the presence of amphibole (Am, RUFF proxy ID: R110203), muscovite (Ms, R040104), quartz (Qtz, R040031), feldspar (Fsp, R040068) and hematite (Hem, R040024).

As shown in Fig. 6, diffractograms from ADC1 subsamples displayed wider and less intense peaks. Considering that XRD analyses were run under the same measurement conditions, including the amount of powdered material, it can be deduced that ADC1 subsamples could contain some secondary phase of low crystallinity. By comparing the position of the detected peaks with XRD reference pattern, both subsamples are mainly composed of quartz with minor amounts of plagioclase and muscovite. In the case of LP sample, hematite signals were detected (33.15 and 35.68 2θ), while UP displayed minor amounts of amphibole.

The overall results gathered from the use of both spectroscopic and diffractometric systems are provided in Table 1.

## Discussion

### Determination of drilled cores composition

As the analytical results presented in this work shown, ADC1 and ADC2 drill cores are characterized by a complex mixture of organic and inorganic compounds. Summarized in Table 1, the mineralogical characterization obtained from the combined use of in-situ and laboratory Raman spectrometers is in good agreement with XRD data. Beside confirming the identification of the main mineral phases (quartz, feldspar and muscovite), the two RLS representative prototypes also detected additional minor compounds, whose concentration was often below the detection limit of XRD (anatase, calcite, amphibole and additional phyllosilicate, depending on the sample). Having in mind the forthcoming ExoMars mission, this result is extremely relevant as it demonstrates that the analytical strategy based on the multipoint Raman analysis of powdered samples could effectively help disclosing the composition of complex mineralogical mixtures.

The two Raman spectrometers, operating under the same operational constraints of the RLS instrument, were able to detect phyllosilicate minerals, which are one of the main scientific targets defined for the ExoMars mission. Indeed, it is well known that phyllosilicates are capable of hosting microorganisms and accumulating biomarkers within their crystalline structure, thus potentially playing a key role in the preservation of life traces on Mars^45^. In fact, the large phyllosilicate deposits detected from orbit at Oxia Planum^46,47^ were one of the main drivers in its selection as the landing site for the *Rosalind Franklin* rover.

The results obtained from the phyllosilicate-bearing samples agree with this thesis, as Raman spectra often presented features corresponding to different organics functional groups. Even though the Raman-based detection of organics in Atacama Desert samples was already achieved in previous works^34^, the great astrobiological relevance of the present research is based on the fact that (1) spectra were collected by Raman systems engineered to mimic the quality of RLS, and (2) drilling sites were remotely selected by the RCC team, who was operating the mission simulation from 11,000 km of distance having no more inputs than the data returned from the rover. The functional groups detected by Raman (including –C≡N, –NH2 and C–(NO2)) are compatible with the presence of nitrogen-fixing microorganisms in the drilled samples. Again, this result fits with previous works presented by Maza et al. 2019, who revealed the presence of six potential nitrogen fixers in the subsurface of the Atacama Desert^48^. Furthermore, it must be noted that LP samples returned the higher number of biomarkers spectra, suggesting that the microbial activity in the subsoil (below 15–20 cm of depth) is higher than in the surface. This gradient in microbial activity may be due to the fact that more favourable conditions for life proliferation can be found at higher depths (e.g., higher water content and lower exposure to UV radiation). Knowing that the analysis of subsoil samples is the core strategy for the ExoMars mission to detect traces of life on Mars, the Raman results here described are extremely promising as they confirm its efficacy.

### Evaluation of RLS representative prototypes

By participating to the ExoFiT trial, the RLS team could evaluate advantages and disadvantages provided by the use of the RLS representative prototypes in ExoMars-related studies. Even though there are numerous studies evaluating the capabilities of the RLS ExoMars Simulator, this is the first work presenting Raman data gathered from the portable RAD1 system. For this reason, comparing the results of the two instruments could help evaluating the real scientific capabilities of the RAD1. As shown in Table 1, the main mineralogical phases were correctly identified in RAD1 datasets, which results were in perfect agreement with spectra provided by the RLS ExoMars Simulator. However, when evaluating phases in minor proportions, some additional compounds could be detected by the laboratory setup. One of the main reasons is the fluorescence background, being more intense in spectra obtained with RAD1 at the LCC than it is in the laboratory ones. In the case of ADC2 subsamples, those containing a higher concentration of low crystalline phases (according to XRD), the fluorescence background covered almost completely the Raman vibrational features in the spectra. This difference can be justified by the use of different analytical approaches. In the laboratory, spectra fluorescence was minimized by automatically performing laser-induced quenching on each spot of analysis (by using the same algorithm that implements RLS). However, this procedure was not feasible for in-situ analyses due to the mentioned time constraints and the stability problems of the spectrometer (triggered by adverse meteorological conditions).

It should be also noted that, despite the additional time required by florescence quenching, the number of spectra collected by the RLS ExoMars Simulator within the time constraints of the ExoFiT Trial was higher than those gathered by the RAD1 (manually operated). This result highlights that a more efficient characterization could be achieved through analysis automation. Learning from the Atacama trial experience, the RLS team is planning to couple the RAD1 system to a portable XYZ positioner as well as to implement its software with RLS algorithms for automatic multi-point analysis of samples. These improvements will allow to optimize data collection and to ensure a better simulation of the automatic operating mode of the RLS, thus increasing the scientific relevance of in-situ Raman studies of terrestrial analogue sites.

### Considerations for the ExoMars mission

Using the data provided by the rover, the controlling team at the RCC the rover was capable of analysing the surrounding environments and identifying areas of scientific interest (PanCam and ISEM), investigating the textural features of the surface (CLUPI), determining the stratigraphy of the subsoil (WISDOM), extract drill cores (ExoMars drill emulator) and analysing their composition (RLS representative prototypes). Strictly focusing on Raman operations (the logistical and engineering challenges faced during the trial will be presented in a specific work), focusing on Raman operations (the logistical and engineering challenges faced during the trial will be presented in a specific work), the time frame dedicated to the spectroscopic analysis of drilled cores was found to be too narrow to achieve the number of spectra established for the nominal operation of RLS on Mars (between 20 and 39). As summarized in Table 1, the ability to detect minor or trace compounds of great scientific relevance (in this case of study, phyllosilicates and organics functional group) increases with the number of analysed spots per sample. In this sense, the RLS ExoMars Simulator missed the identification of the organic functional groups detected by RAD1 in sample ADC2-UP, this particular case evidences that more than 39 spectra per sample could be sometimes needed. Knowing the RLS will work in combination with MicrOmega, the additional information provided by the IR spectral images could be used to plan more targeted Raman analysis during real operations (for the ExoFiT trial the analysed spots were randomly selected), thus increasing the chances of detecting organics on Mars. However, if a scientifically interesting sample is collected from the Martian subsoil, the chances of detecting potential biomarkers could be increased by performing more than one cycle of spectroscopic analysis. Indeed, this procedure could help optimizing the use of the 32 single-use ovens equipped by MOMA to run GCMS analysis, thus enhancing the possibilities to fulfil the main objective of the mission.

## Conclusions

During the Atacama ExoFiT test a complex series of operations, starting with the descent of the rover from the landing platform and ending with the extraction and analysis of drilled cores, were successfully carried out. Focusing on the analytical characterization of subsoil samples, the RLS representative prototypes demonstrated the key role that Raman spectroscopy could play in the fulfilment of the ExoMars mission objectives. By simulating the operational constraints of the RLS, the instruments used in this exercise disclosed the complex mineralogical composition of the samples, providing results qualitatively comparable to those obtained by a laboratory XRD system. In addition to the inorganic matrix, Raman spectrometers also detected several additional signals that could be assigned to biomarkers. In preparation of the upcoming ExoMars mission, this result confirms the capabilities of Raman spectroscopy, which was able to detect extremophilic microorganisms potentially colonizing the subsurface of Martian-like environments. Similar results on Mars would help in the selection of geological samples to be analysed by MOMA. In spite of the promising results, the comparison between RLS ExoMars Simulator and RAD1 data from sample ADC2-UP suggests that the nominal number of spots per sample the RLS will be nominally analyse on Mars (between 20 and 39) may not be sufficient to ensure the detection of trace compounds potentially present in the sample. This is why the ExoMars mission foresees an unprecedented cooperative approach (combined science), by which the instruments of the analytical laboratory will be able to analyse the same spot of the samples. More specifically, this capability will allow RLS to dedicate part of its operation to the analysis of sample spots previously identified by MicrOmega as regions of interest. Nevertheless, if during operations a sample of Martian subsoil reveals itself to be of high scientific interest, the possibility of running an additional cycle of combined MicrOmega-RLS analysis should be considered. Attending to the lessons learnt from the Atacama ExoFiT test, and recognizing the value of mission simulations in preparation for the ExoMars mission, the RLS team is planning to perform improvements (both hardware and software) of the portable RLS representative prototype, aiming to increase the scientific relevance of in-situ Raman studies of terrestrial analogue sites.
