# Author Correction: ExoFiT trial at the Atacama Desert (Chile): Raman detection of biomarkers by representative prototypes of the ExoMars/Raman Laser Spectrometer

**DOI:** 10.1038/s41598-021-88997-9

**Published:** 2021-04-28

**Authors:** Marco Veneranda, Guillermo Lopez-Reyes, Jesus Saiz, Jose Antonio Manrique-Martinez, Aurelio Sanz-Arranz, Jesús Medina, Andoni Moral, Laura Seoane, Sergio Ibarmia, Fernando Rull

**Affiliations:** 1 Department of Condensed Matter Physics, Crystallography and Mineralogy, Univ. of Valladolid, Spain, Ave. Francisco Vallés, 8, 47151 Boecillo, Spain; 2 National Institute for Aerospace Technology (INTA), Torrejón de Ardoz, Spain

Correction to: *Scientific Reports* 10.1038/s41598-021-81014-z, published online 14 January 2021

This Article contains errors in Figure 3 and Figure 4, where the Figures are duplications of Figure 2. The correct Figure 3 and Figure 4 appear below as Figure 1 and Figure 2.

**Figure 1:**
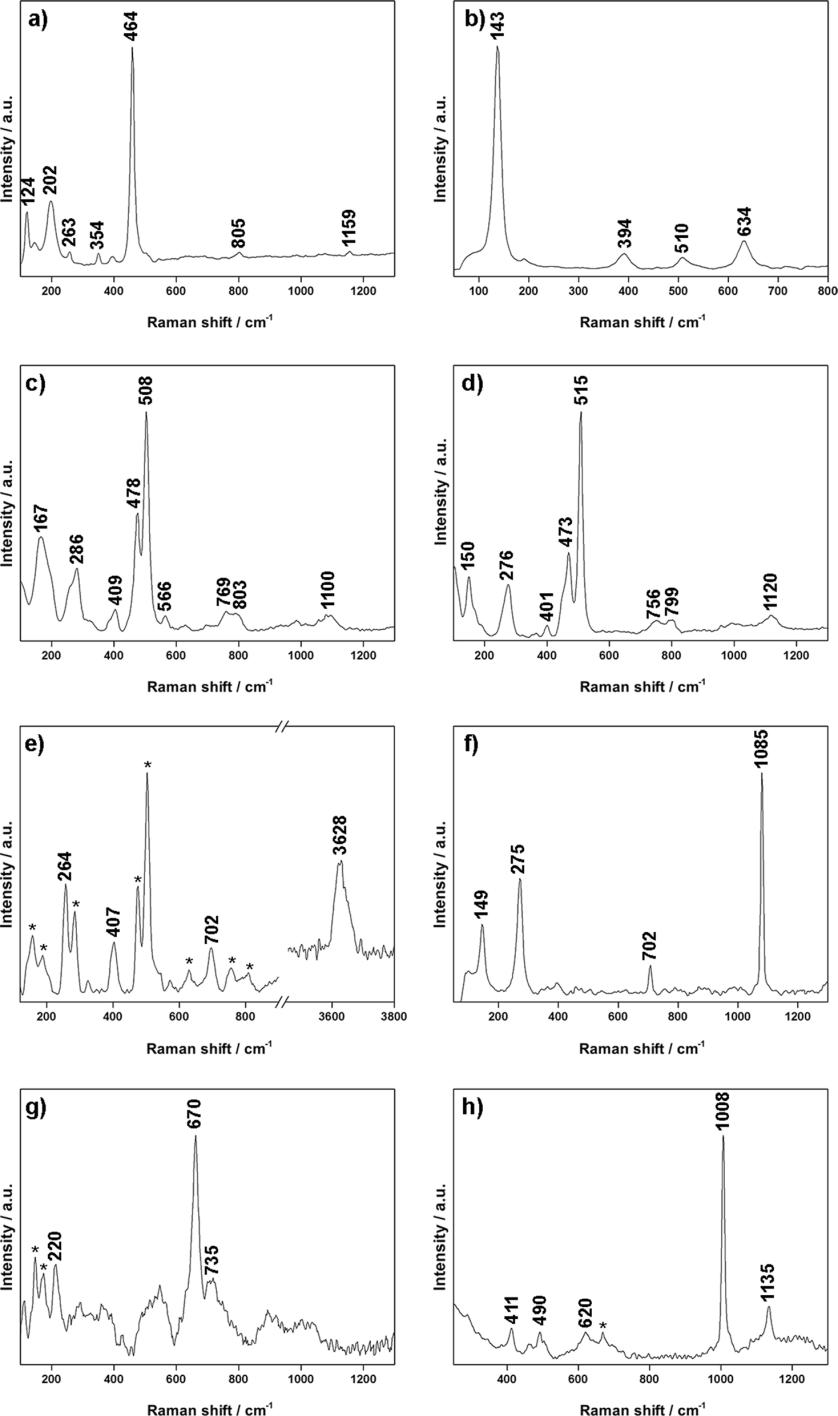
Characteristic Raman spectra of (**a**) quartz, (**b**) anatase, (**c**) plagioclase, (**d**) k-feldspar, (**e**) mica, (**f**) calcite, (**g**) hornblende and (**h**) gypsum, collected in the laboratory by the RLS ExoMars Simulator (532 nm). The baseline of spectra e, g and h has been corrected using dedicated IDAT/SpectPro tools. Raman signals proceeding from additional compounds are labelled with an asterisk.

**Figure 2:**
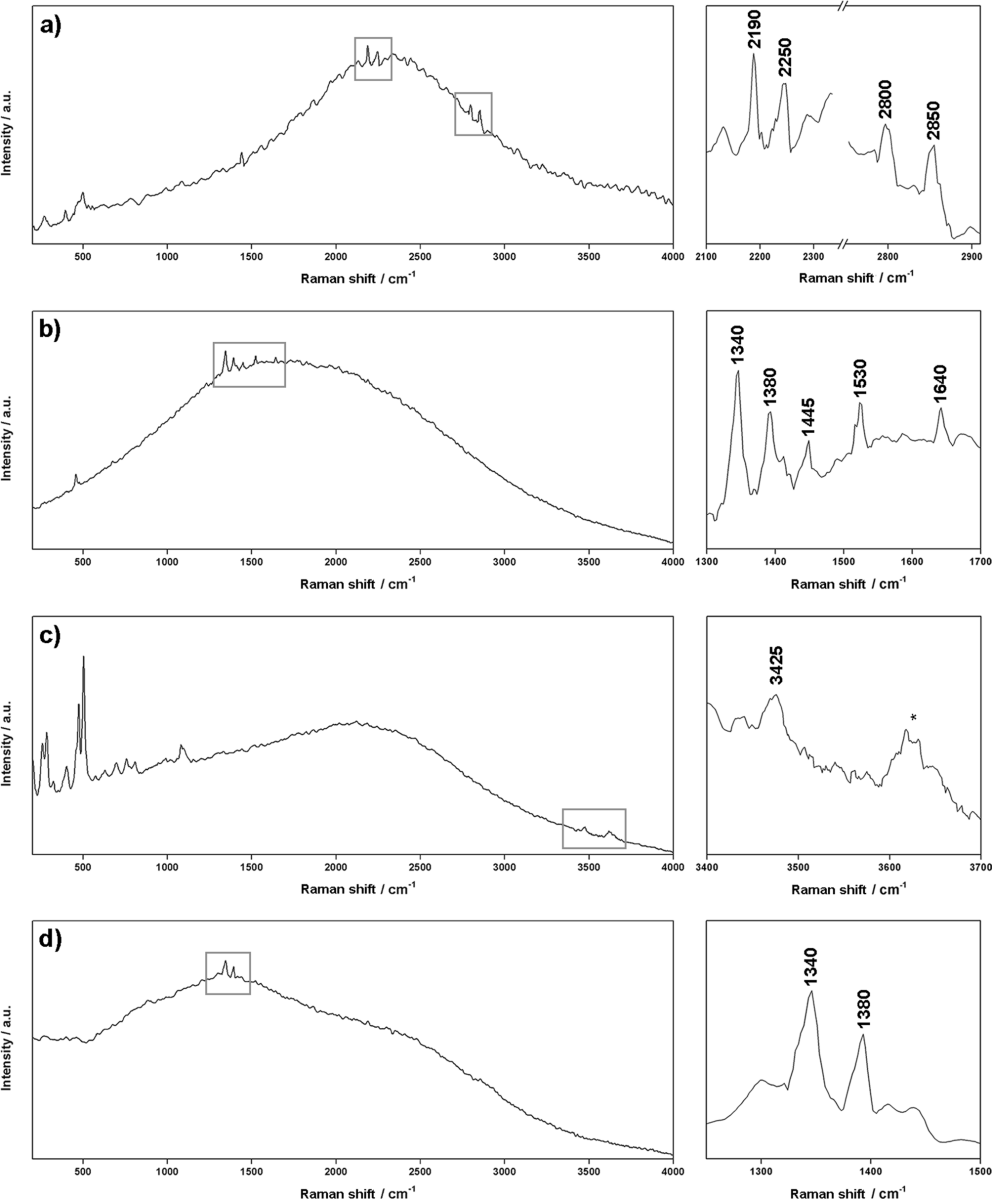
Characteristic Raman spectra of organic compounds collected in the laboratory by the RLS ExoMars Simulator (532 nm). All spectra have been smoothed using dedicated IDAT/SpectPro tools.

